# Supplementary material for: From sequence to enzyme mechanism using multi-label machine learning
Source: BMC Bioinformatics. 2014 May 19;15:150. doi: 10.1186/1471-2105-15-150 (PMC4229970; doi:10.1186/1471-2105-15-150)
Supplement: Additional file 2 — Java code of ml2db. Additional file ml2db_code.tar.gz contains the Java source code to run the multi-label machine learning experiments and save the results to database. The code’s Javadoc is included. [file 1471-2105-15-150-S2.zip › additional file 2/ml2db/ml2db/doc/mulan/data/ConditionalDependenceIdentifier.html]

ConditionalDependenceIdentifier


JavaScript is disabled on your browser.


- Overview
- Package
- Class
- Use
- Tree
- Deprecated
- Index
- Help

- Prev Class
- Next Class

- Frames
- No Frames

- All Classes

- Summary:
- Nested |
- Field |
- Constr |
- Method

- Detail:
- Field |
- Constr |
- Method


mulan.data

## Class ConditionalDependenceIdentifier

- java.lang.Object
- - mulan.data.ConditionalDependenceIdentifier

- All Implemented Interfaces:
  :   java.io.Serializable, LabelPairsDependenceIdentifier

  ---

    

  ```
  public class ConditionalDependenceIdentifier
  extends java.lang.Object
  implements LabelPairsDependenceIdentifier, java.io.Serializable
  ```

  A class for identification of conditional dependence between each pair of labels. The conditional dependence between each pair of labels
  is estimated by evaluating the advantage gained from exploiting this dependence for binary classification of each one of the labels.
  Following the definition of conditional independence, for two conditionally independent labels, predictions of a label by probability-based classification
  models trained once on a regular features space and second on the features space augmented by the second label should be at least very similar. For this
  estimation two binary classifiers are trained and their accuracy is estimated using k-fold cross-validation. If the accuracy of the model trained on the
  features space augmented by the second label is significantly higher, the labels are considered conditionally dependent. The statistical significance of
  the difference between both classifiers is determined using a paired t-test. This procedure is performed for all possible label pairs considering the label
  order in the pair . Among the two pairs with the same labels, the pair with maximal t-statistic value is added to the resulting list of dependent pairs. Finally,
  the resultant label pairs are sorted according to their t-statistic value in descending order (i.e., from the most to the least dependent pairs).

  Version:
  :   30.11.2010

  Author:
  :   Lena Chekina (lenat@bgu.ac.il)

  See Also:
  :   Serialized Form

- - ### Constructor Summary

    Constructors

    Constructor and Description

    `ConditionalDependenceIdentifier(weka.classifiers.Classifier classifier)`

    Initializes a single-label classifier used to perform dependence test between labels and a caching mechanism for reusing constructed models.
  - ### Method Summary

    Methods

    | Modifier and Type | Method and Description |
    | --- | --- |
    | `LabelsPair[]` | `calculateDependence(MultiLabelInstances mlInstances)` Calculates t-statistic value for each pair of labels. |
    | `double` | `getCriticalValue()` Returns a critical value |
    | `int` | `getNumFolds()` |
    | `int` | `getSeed()` |
    | `void` | `setCriticalValue(double criticalValue)` |
    | `void` | `setNumFolds(int numFolds)` |
    | `void` | `setSeed(int seed)` |

    - ### Methods inherited from class java.lang.Object

      `equals, getClass, hashCode, notify, notifyAll, toString, wait, wait, wait`

- - ### Constructor Detail


    - #### ConditionalDependenceIdentifier

      ```
      public ConditionalDependenceIdentifier(weka.classifiers.Classifier classifier)
      ```

      Initializes a single-label classifier used to perform dependence test between labels and a caching mechanism for reusing constructed models.

      Parameters:
      :   `classifier` - - a single-label classifier used to perform dependence test between labels.
  - ### Method Detail


    - #### calculateDependence

      ```
      public LabelsPair[] calculateDependence(MultiLabelInstances mlInstances)
      ```

      Calculates t-statistic value for each pair of labels.

      **Specified by:**
      :   `calculateDependence` in interface `LabelPairsDependenceIdentifier`

      Parameters:
      :   `mlInstances` - the `MultiLabelInstances` dataset on which dependencies should be calculated

      Returns:
      :   an array of label pairs sorted in descending order of the t-statistic value


    - #### setCriticalValue

      ```
      public void setCriticalValue(double criticalValue)
      ```

      Parameters:
      :   `criticalValue` -


    - #### getCriticalValue

      ```
      public double getCriticalValue()
      ```

      **Description copied from interface: `LabelPairsDependenceIdentifier`**

      Returns a critical value

      **Specified by:**
      :   `getCriticalValue` in interface `LabelPairsDependenceIdentifier`

      Returns:
      :   critical value


    - #### getSeed

      ```
      public int getSeed()
      ```

      Returns:


    - #### setSeed

      ```
      public void setSeed(int seed)
      ```

      Parameters:
      :   `seed` -


    - #### getNumFolds

      ```
      public int getNumFolds()
      ```

      Returns:


    - #### setNumFolds

      ```
      public void setNumFolds(int numFolds)
      ```

      Parameters:
      :   `numFolds` -


- Overview
- Package
- Class
- Use
- Tree
- Deprecated
- Index
- Help

- Prev Class
- Next Class

- Frames
- No Frames

- All Classes

- Summary:
- Nested |
- Field |
- Constr |
- Method

- Detail:
- Field |
- Constr |
- Method
